# The function of chemical folic acid in calibration methods and neurodevelopmental disorders

**DOI:** 10.3389/fchem.2024.1355848

**Published:** 2024-02-22

**Authors:** Ziqi Zhou, Meng Wang, Qiongli Fan, Yan Zhao, Nianrong Wang

**Affiliations:** ^1^ Department of Children Healthcare, Chongqing Health Center for Women and Children, Chongqing, China; ^2^ Child and Adolescent Department of Chongqing Mental Health Center, Chongqing, China; ^3^ Department of Pediatrics, Xinqiao Hospital, Army Medical University, Chongqing, China

**Keywords:** functions, folic acid, biological structure, detections, neurodevelopmental disorder

## Abstract

Functional molecules have been attracting increasing attention in environmental and physiological studies. In particular, folic acid (FA) could be considered a key factor in estimating, adjusting, and making decisions in the treatment of neurodevelopmental disorders. It promotes the general significance and conceptual for considering FA molecular scientific research detections, which implies related advancement in both of biological structure and detection methods. Among these applications, the FA molecule acts as a coenzyme that incorporates carbon atoms and synthesizes purines and pyrimidines. Therefore, the calibration method has real applications and can be used as a sensing platform and for detection approaches, which conveys the internal relationship between the FA molecule and physiological characterization. This mini review briefly discusses multiple FA application fields and detection pathways and could supplement their utilization in anticipation of the onset of disease.

## 1 Introduction

As an ordinary organic molecule, folic acid (FA) has been utilized and investigated in biological applications due to its structural specificity, functional ability, psychophysiology estimation, and target therapies (Gazzali et al., 2016). Almost all of the FA in living organisms is used to constitute coenzyme A, which is involved in the transfer of acyl groups in substance metabolism. In the fabrication of functional composites and polymer reactions, for example, FA exhibits a corresponding influence on the construction of enzymes, and the product of its metabolism is related to neurodevelopmental disorders (Gao et al., 2016; Murray et al., 2018; Lintas, 2019). A few studies have referred to the utilization of FA in current research areas. They not only proved that it can be used as a specific physiological parameter but also as a special functional group for the detection of special polymers and the construction of functional structures. Therefore, the FA molecule can be treated as a target agent in well-organized tissues or constructions due to its natural properties, which indicate the free radical scavenging behavior of FA. Previous studies have suggested the possibility that FA exhibits antioxidant activity (Joshi et al., 2001). According to natural characterizations, it conveyed the “nutritional stability” of FA can be considered as main components in organism cleavage products (O’broin et al., 1975). In other words, the complexes in FA analytes were monitored through analysis detection approaches, such as the function of the enzyme before and after natural metabolism (Rosenberg, 2012; Abbasi et al., 2023; Khatamian et al., 2023). Additionally, FA can be used as a conjunction linker in fabricating different functional structures by making different structures (Nguyen et al., 2019; Amirishoar et al., 2023). In terms of different compositions, the existing mechanism was attributed to physical adsorption and bonding rich influences. Electrostatic interactions enhanced these linkage functions, and the corresponding full structures were as well organized as possible. Additionally, many studies have referred to the neurological diagnosis while FA molecules were mediated in different physiological index characterizations (Falah et al., 2020; Zehra and Khan, 2020; Gamboa Gonzales et al., 2023). In these studies, it was necessary to track down the function of FA in various physiological processes so that the monitoring and sensing of its characterizations could be considered as an essential factor. In neurodevelopmental disorders, for example, FA could offer an acceptable way to avoid symptoms associated with a poor neural tube (De La Fournière et al., 2020; Wald, 2022) and improve medical symptoms related to autism (Tan et al., 2020; Hoxha et al., 2021). Therefore, the various functional roles of the cognitive FA molecule can provide strong support for making full use of its molecular properties. FA is an essential water-soluble vitamin in the human body and plays an important role in its metabolism. Clinically, total folic acid is mainly detected using the competitive protein binding method, but the results are not comparable between different detection systems. In this mini review, we mainly discuss the function of FA in the calibrating method, which was built in different functional groups or constructions through optical or chemical measurement. The main sensing function of FA was tracked during physiological processes and related external phenomenon.

## 2 The function of FA in sensing approaches and making functional constructions with continuous optical characterizations

**FIGURE 1:**
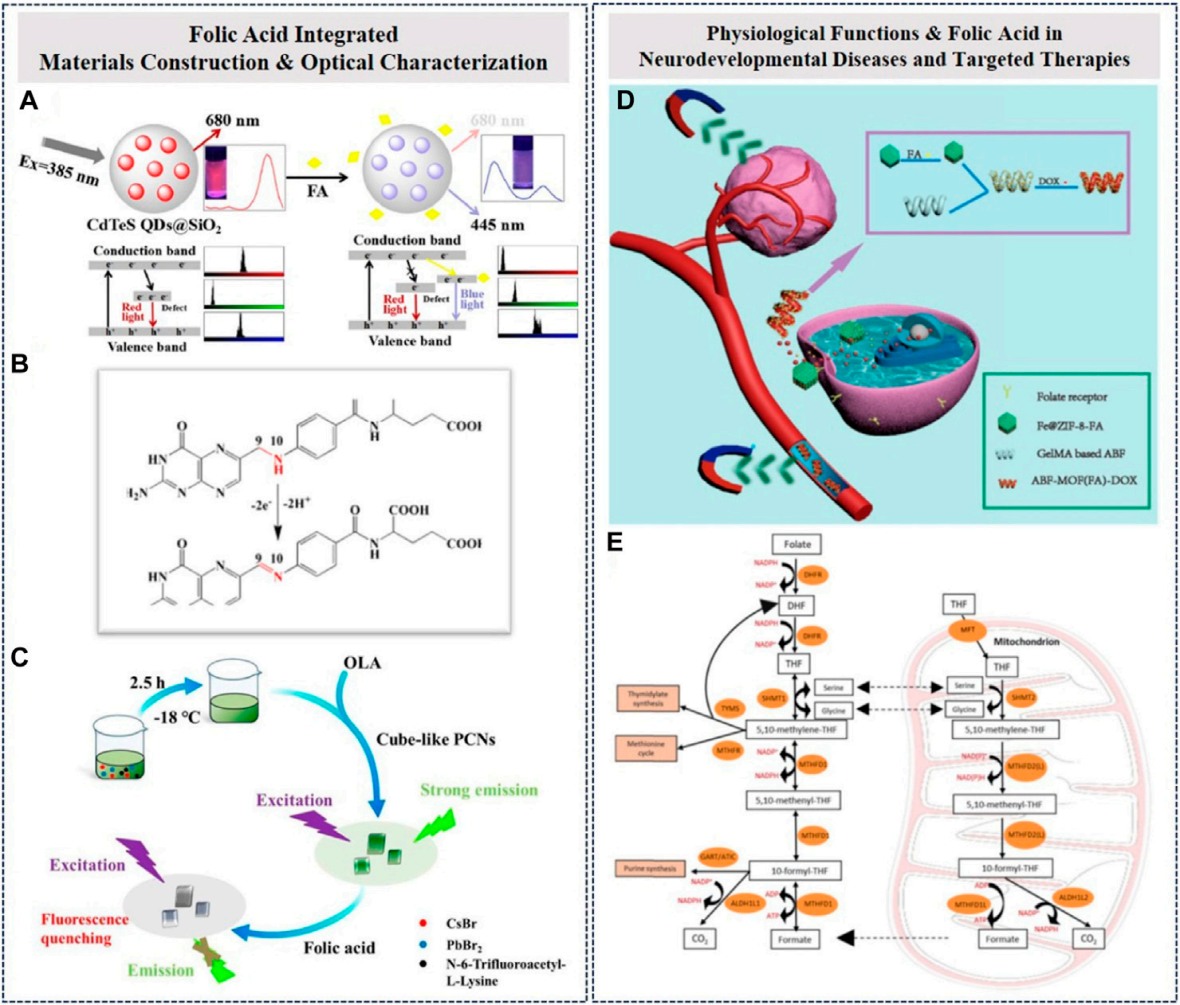
**(A)** Visual detection of folic acid (FA) based on silica-coated CdTeS quantum dots in serum samples (Qian et al., 2018). **(B)** The full structure of FA derivative in sensing platforms undergoing an electron transferring phenomenon (Li and Yan, 2019). **(C)** Selective detection of FA using a water-stable fluorescent CsPbBr_3_/Cs_4_PbBr_6_ perovskite nanocrystal probe (She et al., 2023). **(D)** Magnetic microrobots with FA targeting for drug delivery and induced molecular recognition. **(E)** FA metabolism with a re-emerging therapeutic target in hematological cancers and physiological characterization. Reproduced from Yang et al. (2021) with the permission of the Materials Research Bulletin. Reproduced from Zhou et al. (2022) with the permission of Analytical and Bioanalytical Chemistry. Reproduced from She et al. (2023) with the permission of Chemosensor. Reproduced from Ye et al. (2023) with the permission of Cyborg and Bionic Systems. Reproduced from Zarou et al. (2021) with the permission of Leukemia.

Recently, to improve the analytical performance and electron kinetics of electrochemical biosensors, nanomaterials have been incorporated into the design process of biosensors (Akbar et al., 2016; Di Tinno et al., 2021). Several FA sensors have been reported through the use of biological molecules, such as amino acids and proteins. For sensing applications, precious metal materials were mainly utilized in fabricating optical sensors for FA detection based on fluorescence properties. It focus on the specificity in FA structure and the particular sensor signals can be monitored in optical devices and biological platforms. The detection of FA in each biological application or chemical reactions could be presented in different forms and measurements. It is just the supplements and synergistic reaction in sensing works. Here, gold cluster-based FA biosensors must be designed to analyze the fluorescence and absorbance spectra of the gold clusters in the presence and absence of FA. The mechanism of the fluorescence quenching of bovine serum albumin (BSA)-modified gold clusters by FA was investigated. At a pH of 7.4, the fluorescence quenching was well suited to the Stern–Volmer equation, with a wide linear response in the concentration range (120.0 ng/mL to 33.12 μg/mL, and a LOD of 18.3 ng/mL). Furthermore, the sensor was applied for the detection of FA in pharmaceutical samples (Hemmateenejad et al., 2014). As been illustrated biomarker, in diagnosing early diseases characterization, it implies the practical significance to explore an appropriate biomaterials as useful tools in sensing FA. Herein, a visual fluorescent probe was creatively designed by wrapping silica shell (SiO_2_) on the surface of CdTeS quantum dots (CdTeS QDs@SiO_2_, Figure 1A). The color change of the samples could be easily recognized by the naked eye and was analyzed based on the red, green, and blue (RGB) values obtained using image software (Yang et al., 2021). Benefiting from the synergistic effect of different functional materials in the detection process, a perovskite nanocrystal (PNC) probe can also be used to construct probe structures for FA molecular detection (Figure 1B). The interaction between the PNCs and small biological molecules was investigated and the results indicated that the fluorescence of the PNCs could be selectively quenched by FA. The quenching rate has an exact linear relationship with the concentration of FA. The mechanism of the interaction between the PNCs and FA was discussed, and the reliability of the method for real sample detection was also verified by the standard method. The method proposed here, using a fluorescence PNC probe, provided a simple alternative strategy for detecting FA that will play an important role in biochemical analysis (Qian et al., 2018; Li and Yan, 2019; She et al., 2023). It also conveyed the functional construction with continuous optical characterization about FA detection. However, the related examples use the successful experiences of others to be treated available substitutions. Under various conditions, the FA molecule could be treated as a target in biomedical or bioengineering issues due to its marker attributes and metabolic properties (Figure 1C). Attributed to the basic principle of FA biosensors (Zhou et al., 2022), these electroanalytical devices can be used in the detection of various diseases, such as cancer (Krishnapriya et al., 2023), cardiovascular diseases (Wald et al., 2018), neural tube defects (Berry, 1999), and megaloblastic anemia (Chanarin et al., 1962); therefore, it is a hot topic in sensing industries. As mentioned in existed characterizations, the main principles is obviously defined in each sensing platform. It expresses the main factor is the functions of FA structure (various activated states). It directly assists and consists the sensing progresses. It reflects key principles, such as the molecule reaction between functional group crosslinking phenomena. Similarly, the different statuses of FA in detection methods conveyed the related responses in biological or physiological characterizations.

## 3 The physiological characterizations of FA in neurodevelopmental diseases and targeted therapies

There was a similar case in neurodevelopmental disorders, the content of FA molecule plays key role in the disease adjudgement (Chatzi et al., 2012; Bjork et al., 2017). In terms of physiological features, FA metabolism disorder can easily lead to psychiatric disorders. Abnormal FA metabolism leads to disorders in the absorption or intake of FA, resulting in a deficiency in the whole body. From the clinical manifestations, FA metabolism dysfunction leads to skin changes, oral ulcers, depression, loss of appetite, nausea, vomiting, neurasthenia, giant erythrocyte anemia, and other manifestations. All these characterizations could influence the related behaviors in daily life. Possible methods of monitoring FA should be determined in real diagnosis. The current method of diagnosis includes the diagnosis of FA metabolic disorder, which was carried out mainly through genetic testing, serum FA level testing, combined with clinical manifestations, CT, MRI and other examinations, and disease diagnosis (Romano et al., 1995). The diagnosis of mental illness is mainly carried out through clinical manifestations, the medical history collection, and psychological evaluation. Additionally, there have been several studies of the physiological metabolism that referred to the function of FA in targeted therapies, such as FA magnetic guide targeting by microrobots (Zarou et al., 2021; Ye et al., 2023). The microrobots can successfully navigate through the magnetic field and gather around the lesion site due to the closed drug-carrying system of FA (Figure 1D). This technique was developed in drug loading and drug delivery systems (Quan et al., 2018). Similarly, based on existing studies, cognitive FA-mediated one-carbon metabolism and its importance at the cellular level are of practical significance for the subsequent development of targeted FA metabolism for the treatment of blood cancer (Ruiyi et al., 2020). Furthermore, it was one essential appointment for the exploration of therapeutic strategies to overcome the limitations of traditional antifolate strategies (Figure 1E). In this situation, the FA molecule becomes the main carrier of supporting carbon elements that form various living substances. The organism-specific expression derived from the living matter formed in the turnover process becomes the main mechanism constituting the molecular physiological characterization of the FA molecule. Currently, the essential clues in different physiological characterizations have yet to be clearly determined with regard to small molecule metabolites. In all these processes, FA was regarded as the only target and was well characterized in different work schedules. Toward the FA application works, it is necessary to know biophysiological activity through first carbon transfer in natural works, in the carbon units transferring process, which gives directed assistance in synthesis of biological molecules. We can make full use of this mechanism, complete the qualitative analysis of the products before and after the reaction, and then identify the effect of the amount of different folic acid molecules on life activities. Therefore, how to realize the quantitative determination of FA in synergistic drug loading and the physiological metabolism on the basis of qualitative analysis has undoubtedly become the main issue for subsequent studies.

## 4 Conclusion and outlook

This mini review has described the recent advancements in FA-based issues and referred to the detection methods and physiological characterization. Similarly, FA should be monitored and detected in various forms through analytical methods and instrumental actions. An initial estimation suggests the FA could play a linkage role in the construction of different functional structures due to its molecular architectural features. After being combined with organic or inorganic contents, the original optical properties of the full structure could change the physical or chemical properties, which also reflected the mechanism of the actions of FA. Owing to the changes in the optical measurements, the changes visible to the naked eye and fluorescence could indirectly prove that FA is present. In this case, different detection methods can be merged to improve the detection and sensing capacity. However, in addition, the influence of FA on physiological appearance was related to neurodevelopmental diseases in the form of physiological traits and physiological metabolites. The physiological characterizations of FA in neurodevelopmental diseases and targeted therapies implied that the function of FA in sensing approaches needs to be investigated, as suggested in the first section. In terms of its application in physiological fields, FA was treated as a target for determining the possibility of neurodevelopmental diseases, not only as a target molecule but also as one of the factors in the potential treatment in cancer therapies. It should considered there have enough significance while FA based functional construction is utilizing in clinical diagnosis. In which, the understanding in characteristics of FA is mainly attributed in induced properties and selectivity. In addition, this implied the use of optical probes for precise analysis at cellular and tissue levels according to FA doped with specific contents, providing more accurate diagnostic and therapeutic tools in clinical medicine. By recognizing the fundamental property, these different types and forms in FA-based characterization play an important role in the early diagnosis of diseases, individualized therapy, and life-accessible research.
